# Methods for Coding Tobacco-Related Twitter Data: A Systematic Review

**DOI:** 10.2196/jmir.7022

**Published:** 2017-03-31

**Authors:** Brianna A Lienemann, Jennifer B Unger, Tess Boley Cruz, Kar-Hai Chu

**Affiliations:** ^1^ Department of Preventive Medicine Keck School of Medicine University of Southern California Los Angeles, CA United States

**Keywords:** tobacco, Internet, social marketing, review

## Abstract

**Background:**

As Twitter has grown in popularity to 313 million monthly active users, researchers have increasingly been using it as a data source for tobacco-related research.

**Objective:**

The objective of this systematic review was to assess the methodological approaches of categorically coded tobacco Twitter data and make recommendations for future studies.

**Methods:**

Data sources included PsycINFO, Web of Science, PubMed, ABI/INFORM, Communication Source, and Tobacco Regulatory Science. Searches were limited to peer-reviewed journals and conference proceedings in English from January 2006 to July 2016. The initial search identified 274 articles using a Twitter keyword and a tobacco keyword. One coder reviewed all abstracts and identified 27 articles that met the following inclusion criteria: (1) original research, (2) focused on tobacco or a tobacco product, (3) analyzed Twitter data, and (4) coded Twitter data categorically. One coder extracted data collection and coding methods.

**Results:**

E-cigarettes were the most common type of Twitter data analyzed, followed by specific tobacco campaigns. The most prevalent data sources were Gnip and Twitter’s Streaming application programming interface (API). The primary methods of coding were hand-coding and machine learning. The studies predominantly coded for relevance, sentiment, theme, user or account, and location of user.

**Conclusions:**

Standards for data collection and coding should be developed to be able to more easily compare and replicate tobacco-related Twitter results. Additional recommendations include the following: sample Twitter’s databases multiple times, make a distinction between message attitude and emotional tone for sentiment, code images and URLs, and analyze user profiles. Being relatively novel and widely used among adolescents and black and Hispanic individuals, Twitter could provide a rich source of tobacco surveillance data among vulnerable populations.

## Introduction

As Twitter has grown in popularity to 313 million monthly active users [1], researchers have increasingly been using it as a data source for tobacco-related research. Twitter is a microblogging platform where users have 140 characters to share thoughts, jokes, information, images, and URLs (ie, Web addresses). Twitter posts (ie, tweets) are in real time and often public, with the potential to reach a wide audience. Users can retweet or share tweets with others, which can cause tweets to spread to large numbers of users (“go viral”). Posts can be seen when users follow each other or search for specific terms (eg, #vape). Hashtags signify a topic for users to participate in the conversation. Antitobacco campaigns may use a hashtag to start a conversation about the harms of tobacco, for example, the Truth Initiative account, @truthinitiative, promotes the use of #tobaccofreegen in the user description [2]. Similarly, the tobacco industry and independent manufacturers can use Twitter to advertise their products. Imperial Brands uses such an approach when it promotes the electronic cigarette (e-cigarette) blu through its account, @blucigs, with the hashtag #JustYouAndblu in the user description and messages to engage with Twitter users [3]. Furthermore, social media can be used to counter antitobacco campaigns. When the anti–e-cigarette campaign Still Blowing Smoke [4] was released by the State of California on television and Facebook, the pro–e-cigarette campaign Not Blowing Smoke [5], developed by pro-vaping groups, countered by taking the Twitter handles @StillBlwngSmoke [6] and @NotBlwngSmoke [7] to challenge the California campaign’s messages [8].

Tobacco-related tweets can reach a relatively young and ethnically diverse audience. Pew Research Center studies have found that, in the United States, a third of teenagers use Twitter [9], while 20% of adults have accounts with the majority being younger than 50 years [10]. Twitter is especially popular among girls aged 15 to 17 years with 49% having accounts [9]. Similarly, 45% of black, 34% of Hispanic, and 31% of white adolescents use Twitter [9]. Comparably, a larger proportion of black (28%) and Hispanic (28%) than white (20%) adult *Internet users* also use Twitter [10]. Therefore, tobacco conversations on Twitter, whether pro or anti, may be particularly likely to reach these populations.

Research utilizing Twitter data is fairly novel without established standards across studies. Thus, it could be advantageous to establish what methods are being used and their strengths and weaknesses. Standards for reporting social media data are needed to be able to compare methods and results across studies [11]. This review focuses specifically on the methodology of tobacco-related studies that code Twitter data categorically by examining data collection methods, coding methods, and coding categories. It addresses the questions, “What methodologies are used to categorically code tobacco-related Twitter data” and “What recommendations can be made for future studies?”

## Methods

### Data Sources

**Figure 1:**
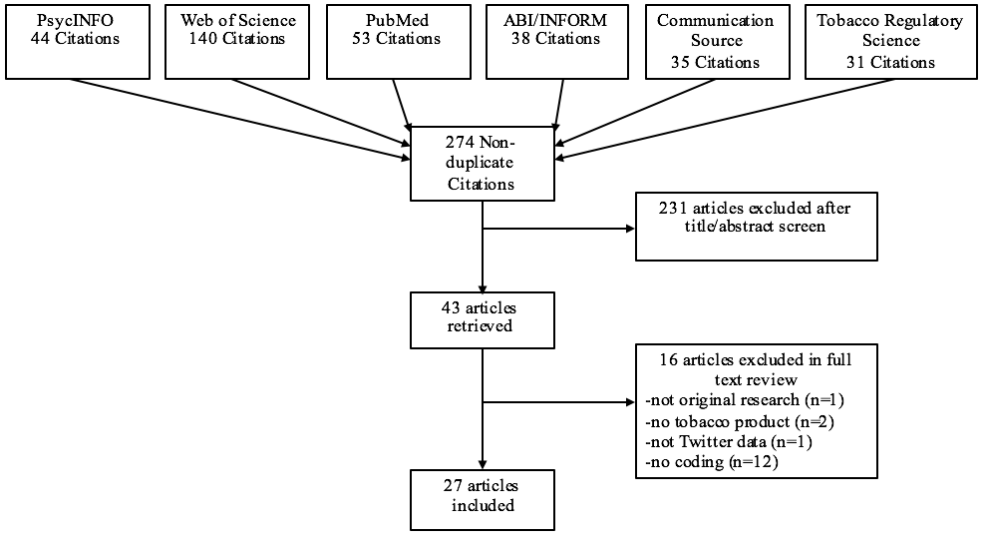
PRISMA (Preferred Reporting Items for Systematic Reviews and Meta-Analyses) diagram of articles included in the systematic review.

A literature search was conducted in July 2016 using the databases PsycINFO, Web of Science, PubMed, ABI/INFORM, Communication Source, and the journal Tobacco Regulatory Science. Searches included a Twitter term and a tobacco term: (Twitter OR tweet) AND (tobacco OR nicotine OR...) (Table 1). Tobacco terms were selected based on an article on noncigarette tobacco products [12] and the US Food and Drug Administration’s (FDA) article, *Recognize Tobacco in Its Many Forms* [13]. Searches were limited to peer-reviewed journals in English published from January 2006 to those available in July 2016. The beginning date was selected because Twitter was launched in 2006. The initial search produced 274 nonduplicate articles (Figure 1).

**Table 1:** Tobacco search terms.

Search term^a^	Tobacco products covered by search term
tobacco	Tobacco, smokeless tobacco, chewing tobacco, dissolvable tobacco
nicotine	Nicotine, electronic nicotine delivery system
cig*	Cigarette, cigar, little cigar, large cigar, cigarillo, electronic cigarette, e-cigarette, e-cig
pipe	Pipe, waterpipe
bidi	Bidi
kretek	Kretek
shisha	Shisha
hookah	Hookah, e-hookah, hookah pen
narghile	Narghile
argileh	Argileh
cheroot	Cheroot
smok*	Smoke, smokeless tobacco, smoking, smoker
chew	Chew, chewing tobacco
snuff	Snuff, dry snuff, moist snuff
snus	Snus
betel quid	Betel quid
gutkha	Gutkha
zarda	Zarda
toombak	Toombak
dissolvable	Dissolvable, dissolvable tobacco
ENDS	ENDS (electronic nicotine delivery system)
vap*	Vape, vaper, vape pen, vaping, vapor

^a^Asterisk (*) represents stemmed words; for example, cig* would capture all words beginning with cig.

### Study Selection

One coder independently reviewed all titles and abstracts and selected 43 articles from the initial 274 that referenced any of the tobacco products and Twitter. Full text of the articles was then reviewed. Explicit inclusion criteria were determined a priori to reduce coder bias when selecting articles. Articles were included in the review if they met the following criteria: (1) original research in a peer-reviewed journal, (2) focused on tobacco, (3) analyzed Twitter data, and (4) coded data categorically (eg, sentiment, theme). If an article was excluded for failing to meet multiple inclusion criteria, it was counted in the higher-order criteria for exclusion. A total of 16 articles were excluded, so 27 articles were included in the review (Figure 1). We excluded 1 article because it was a narrative review rather than original research, 2 articles because they did not address a tobacco product, 1 article because it analyzed Web search results and tobacco control websites rather than Twitter data, and 12 articles because they did not categorically code tobacco-related Twitter data. For example, a study may have tracked changes in the number of tweets over time but not coded the tweets categorically. The 27 included articles ranged from 2011 to 2016. Although there is risk for bias in studies, this review considered all studies that met the inclusion criteria to evaluate the strengths and weakness of all methodological approaches within this domain. The primary focus of this review is the methodology of qualitative studies rather than the outcomes of quantitative studies. Therefore, the risk of publication bias of studies with significant results and selective reporting of significant results is minimal for this type of review compared with a meta-analysis of quantitative outcomes.

### Data Extraction

One author read each article to extract methodological information on data collection (data sources, date collected, tobacco topics, search keyword selection), coding methods (type of coding method, number of coders, number of tweets coded, coded retweets, number of Twitter accounts, followed URLs, coding agreement), and coded categories. This information is presented in detail in Tables 2, 3, and 4 and is summarized in the results.

## Results

### Data Collection Methods

#### Data Sources

In total, 22 of the 27 articles discussed the sources they used for their sample of Twitter messages, while 5 articles did not. Twitter provides 3 primary sources of data: Twitter’s Search application programming interface (API), Twitter’s Streaming API, and Twitter’s Firehose. An additional method is Twitter’s REST API, which allows tracking specific users by their username. One study collected data using Twitter’s REST API.

Twitter’s Search API is free to use and provides a maximum of 3200 past tweets (ie, published in the past 7 days, so it is not in real time) with a limit of 180 searches every 15 minutes [14,15]. Twitter’s Search API was used in 2 studies (Table 2). There are programs developed to interact between Twitter’s Search API and specific analyses programs. For example, twitteR package for R [16], NCapture for NVivo [17], and Social Network Importer for NodeXL, the free add-on for Excel [18], provide access to Twitter’s Search API. A small subset of the studies used these programs: twitteR package for R (n=1), NCapture (n=1), and Social Network Importer for NodeXL (n=1).

More useful to researchers is Twitter’s Streaming API, which provides all tweets related to the search terms up to a limit of 1% of the Twitter database for that time period. If the selected search terms are infrequently used across Twitter (eg, the name of a local tobacco campaign), all tweets related to the search terms will be available. However, if the selected search terms are commonly used, tweets related to those terms will be available up to a limit of 1% of the current Twitter database. Twitter’s Streaming API is free, publicly available data [19]. A total of 5 studies used Twitter’s Streaming API (Table 2). Twitonomy is an analytics tool that accesses Twitter’s Streaming API and offers both free and premium packages for a small fee [20]. One article used Twitonomy.

Providing the greatest access to data, Twitter’s Firehose has real-time access to 100% of Twitter content. Twitter’s Firehose formerly was handled by multiple data providers (eg, Gnip, DataSift, and Topsy). After the acquisition of Gnip in 2014, Twitter transitioned to only allowing access to Twitter’s Firehose through Gnip for a fee from August 2015 onward [21]. A total of 6 articles used Gnip, making it the most common method to collect data (Table 2). There are social listening programs that buy Twitter data from data providers such as Gnip. Radian6 [22], Simply Measured [23], and Sysomos Heartbeat [24] collect data from across social networking sites, blogs, forums, and news sites for a fee. A few articles used these social listening programs to collect data: Radian6 (n=2), Simply Measured (n=1), and Sysomos Heartbeat (n=1).

**Table 2:** Data collection methods.

Article	Date collected	Type of tweets or accounts	Keyword selection^a,b^	Data source	Retrieval precision	Retrieval recall
[8]	March 22 to June 27, 2015	Tweets about the California Department of Public Health “Still Blowing Smoke” media campaign about the harms of e-cigarettes and the pro–e-cigarette campaign “Not Blowing Smoke”	#stillblowingsmoke, stillblowingsmoke, “still blowing smoke”, stillblngsmoke, “still blng smoke”, #notblowingsmoke, notblowingsmoke, “not blowing smoke”, notblngsmoke, “not blng smoke”, @CAPublicHealth	Gnip	97.5%	NR^c^
[25]	May 1, 2013, to May 1, 2014	E-cigarettes	vaping, vape, vaper, vapers, vapin, vaped, evape, vaporing, e-cig*, ecig*, e-pen, epen, e-juice, ejuice, e-liquid, eliquid, cloud chasing, cloudchasing, deeming AND regulation, deeming AND FDA, deemed AND FDA, deem* AND FDA	Gnip	59.23%	NR
[26]	July 1, 2008, to February 28, 2013	E-cigarettes	*55 keywords (only examples reported)*: *general e-cigarette terms* (*eg,* electronic cigarette, eCig), *specific brand names* (*eg,* blu, NJoy, green smoke), *and terms about e-cigarette use* (*eg*, vaping) *Excluded words related to tobacco or drugs (only examples reported)*: marijuana, hookah	Radian6	91%	93%
[27]	December 5, 2011, to July 17, 2012 (15-day intervals)	Tobacco	cig*, nicotine, smok*, tobacco; hookah, shisha, waterpipe, e-juice, e-liquid, vape *and* vaping	Twitter’s Streaming API^d^	57.25%	95%-99%
[28]	January 1 to December 31, 2014	E-cigarettes and smoking cessation	*E-cigarette keywords*: vaping, vaper, vapes, vapers, e-cigarette, e-cig, mod, eGo, mod, RBA, RDA, vape, “vape pen”, “e-hookah”, “e-pipe”, “e-shisha”, “hookah pen”, “vape pipe”, #vape #vapelife #vapor #vapeporn #vapenation #vapestars #vaperazzi #vapingstyle #vaperevolution #vapeswag #vapesirens #vaperscommunity #vapepics #vapesociety #socialvapers #vapefamily #vapefreedom #vapelove #vapers #vapstagram #vapelyfe #vapeshop #vapeon #vapestrong #girlswhovape #alldayvape #adv #vapersoul #VGOD #ecig #ecigarette *Smoking cessation keywords*: quit, stop, “quit smoking”, “stop smoking”, “quit cigarettes”, “smoke less”, “smoking less”, help, NRT, patch, lozenge, spray, gum, nicorette, nicotine, #quit #quitsmoking #quitsmokingcigarettes #Cessationnation *Exclusion keywords*: marijuana, weed, pot, dank, trees, green, cheeba, THC, cannabis, sativa, indica, bud, marihuana, MJ, “mary jane”	Sysomos Heartbeat	NR	NR
[29]	February 1 to April 30, 2014	Blu e-cigarettes’ tweets and retweets	@blucigs	Twitter REST API	NR	NR
[30]	April 12 to May 10, 2014	Hookah or shisha	hookah, #hookah, shisha, #shisha, hooka, #hooka, sheesha, #sheesha	Simply Measured	99.56%	NR
[31]	November 1, 2011, to August 31, 2013	Hookah, cigarettes, and cigars	cigar, cigars, cigarette, cigarettes, hookah, waterpipe, water pipe, shisha, sheesha	Twitter’s Streaming API	NR	NR
[32]	October 4 to November 3, 2010	Tobacco	Smoking, tobacco, cigarette, cigar, hookah, hooka	Twitter’s Search API	NR	NR
[33]	May 1, 2012, to June 30, 2012	E-cigarettes	*Keywords*: e-cigarette, ecigarette, e-cig, ecig *Additional keywords AND “cig” or “cigarette”*: electronic, blu, njoy	Gnip	>99% of a random sample of 500 tweets	NR
[34]	December 6, 2012, to June 20, 2013	Tobacco or cessation price promotion	*Tobacco-related*: cig(s), cigarette(s), smoking, tobacco, blu cigarette, njoy cigarette, ecig, e-cig, @blucig, e-cigarette, ecigarette, from:blucigs*, ecigs, e-cigs, ecigarettes, e-cigarettes, “green smoke”, “south beach smoke”, cartomizer, (atomizer OR atomizers) –perfume*, ehookah OR e-hookah, ejuice OR ejuices OR e-juice OR e-juices, eliquid OR eliquids OR e-liquid OR e-liquids, e-smoke OR e-smokes, (esmoke OR esmokes), eversmoke, “joye 510”, joye510, lavatube OR lavatubes, logicecig OR logicecigs, smartsmoker, smokestik OR smokestiks, “v2 cig” OR “v2 cigs” OR v2cig OR v2cigs, vaper OR vapers OR vaping, zerocig OR zerocigs, cartomizers, Vuse, MarkTen *Price-related*: Coupon(s), Promo(s), Promotions(s), Promotional, Discount(s)(ed), Save, Code(s)	Gnip	56.94%	NR
[35]	July 2014	Slogans for the Dutch health campaign “Smoking is so outdated” (Roken kan echt niet meer)	#rokenkanechtnietmeer [#smokingissooutdated]	Twitter’s Search API	NR	NR
[36]	December 2013	Little cigars	Swisher Sweets, Black & Milds	Twitonomy	67.50%	NR
[37]	September 2012 and January to May 2013	Genetic information on smoking	genetic, smoking	NR	49.1%	NR
[38]	August 2010	Smoking cessation accounts	*Searched for smoking cessation accounts using the following terms*: “quit or stop smoking” or “smoking cessation”	NR	NR	NR
[39]	January 8-15, 2014	Tweets about Chicago Department of Public Health’s e-cigarette Twitter campaign	@ChiPublicHealth	twitteR package for R and NodeXL	NR	NR
[40]	January 2010 to January 2015	E-cigarettes	vape, ecig, ecigarette, vaping, ejuice, vapers, drip AND tip, dripping, eliquid AND flavor, e AND juice, e AND liquid, smoke AND free, off AND cigarettes, ex AND smoker, no AND analogs, I AND quit	NR	NR	NR
[41]	January 2012 to December 2014	E-cigarettes	e(-)cig, e(-)cigarette, electronic cigarette, *etc*	Twitter’s Streaming API	81% to 90.8% for 4 groups of 500 randomly sampled automated tweets	NR
[42]	September to December 2013 and March 2015	E-cigarettes	Electronic-cigarette, e-cig, e-cigarette, e-juice, e-liquid, vape-juice, vape-liquid	Twitter’s Streaming API and Twitter’s Firehose	97.21%	86.63%
[43]	April 21 to October 20, 2014	Blu and V2 e-cigarettes’ tweets and retweets	@blucigs, @v2cigs	NR	100%	NR
[44]	July 7 to 21, 2014	Tweets about the Centers for Disease Control and Prevention’s (CDC) Tips From Former Smokers campaign	#cdctips, CDC AND smoking	Social Network Importer for NodeXL	81.70%	NR
[45]	May 1, 2013, to May 1, 2014	E-cigarettes	vaping, vape, vaper, vapers, vapin, vaped, evape, vaporing, e-cig*, ecig*, e-pen, epen, e-juice, ejuice, e-liquid, eliquid, cloud chasing, cloudchasing, deeming AND regulation, deeming AND FDA, deemed AND FDA, deem* AND FDA	Gnip	59.23%	NR
[46]	March to June 2013	Tobacco control program tweets during the months that the national CDC Tips smoking cessation campaign aired	*Google search for tobacco control programs using the terms “* tobacco program *” and “* quitline *.” If the site included a link to a Twitter account, that* *account was included*.	Radian6	NR	NR
[47]	March 15 to June 9, 2012	Tweets about the CDC’s Tips campaign	*Tobacco behavior*: cig(s), cigarette(s), nicotine, smoke(s), smoker, smoking, tobacco *Tobacco policy*: @cdcgov, @cdctobaccofree, @drfriedencdc, @fdatobacco, @smokefreegov, antitobacco, antismoking, CDC, quitline, quitnow, secondhand+smoke, smokefree, smokefree.gov, tobaccofree *Ad specific*: #cdctips, amputation, amputee, Buerger’s+Disease, heart+attack, hole+neck, hole+throat, lung+cancer, stoma, stroke, throat+cancer *Engagement*: ad, commercial, campaign, PSA	Gnip	78.87%	94%
[48]	February 5-12, 2014	CVS Health-related tweets surrounding the announcement of ending tobacco sales	#cvs, #cvsquits	Twitter’s Streaming API	72.38%	NR
[49]	50 most recent tweets from July 18, 2012	Smoking cessation accounts	*Searched for smoking cessation accounts using the terms*: “quit smoking” *and* “smoking cessation”	NR	NR	NR
[50]	February 23 to April 9, 2015	Exposure to secondhand e-cigarette aerosol	“secondhand vape” OR “secondhand vaping” OR “second-hand vape” OR “second-hand vaping” OR “vape smoke” OR “ecig smoke” OR “e-cig smoke” OR “e-cigarette smoke” OR “vape shs” OR “ecig shs” OR “vape secondhand smoke” OR “vape second-hand smoke” OR “esmoke” OR “e-smoke”	NCapture	NR	NR

^a^Asterisk (*) represents stemmed words; for example, cig* would capture all words beginning with cig.

^b^Words in italics were not keywords used for searches.

^c^NR: not reported.

^d^API: application programming interface.

#### Date Collected

Twitter data were collected across the studies from 2008 to 2015. The time span of Twitter data collected within an individual study ranged from 1 day to 5 years with a median of 14 weeks (Table 2).

#### Tobacco Topics

Among the 27 studies, 41% (n=11) analyzed messages related to e-cigarettes, 19% (n=5) related to other tobacco products, and 22% (n=6) about specific tobacco campaigns (Table 2). The remaining topics included smoking cessation accounts (n=2), tobacco or cessation price promotion (n=1), genetic information on smoking (n=1), and ending tobacco sales at CVS Health (n=1).

#### Search Keyword Selection

Kim and colleagues [11] proposed a framework of three steps to develop and validate search filters. This framework was selected because it provided a standard in which to compare studies. Most of the articles partially fulfilled these steps within the framework. The first step is to develop a search filter. All the articles generated a list of keywords presumably based on expert knowledge of the topic or a systematic search of language related to the topic of interest. However, only 4 articles discussed the process of discarding keywords that returned a high proportion of irrelevant results and adding new keywords as new terms appeared in the returned literature [25-28]. It should be noted that for some studies this process of developing a search filter may be irrelevant because they are coding all tweets from a specific account (eg, coding tweets from the blu e-cigarette account, @blucigs) [29].

Search keyword selection was tailored to the studies’ specific topics (Table 2). To search for e-cigarette Twitter data, variations on e-cigarette (eg, e-cig), vape (eg, vaping), e-liquid (eg, eliquid), and e-juice (eg, ejuice) were common. Some e-cigarette studies also included major e-cigarette companies or brands as key search terms (eg, Njoy). Studies that analyzed Twitter data on specific tobacco campaigns used a variety of tactics such as searching for variations on the campaign name (eg, still blowing smoke), the source of the campaign (eg, CDC), specific features of the campaign ads (eg, lung+cancer), tobacco products and behavior (eg, smoke), and general campaign terms (eg, PSA). Some studies also used a combination of searching for terms with and without hashtags (eg, hookah, #hookah). In 2 studies, marijuana terms (eg, weed) were used as exclusion keywords [26,28].

The second step of the framework is to apply the search filter and split data into retrieved and unretrieved sets. The third step is to assess the search filter on its ability to distinguish between relevant and irrelevant messages [11]. Precision refers to how much retrieved data are relevant, whereas recall refers to how much relevant data are retrieved. Recall is similar to measures of sensitivity. Precision is much less difficult to accurately estimate than recall because recall requires conclusions to be drawn about the tweets that were not retrieved. A precision score of 100% means that all retrieved data were relevant, while a recall score of 100% means that all relevant data were retrieved. However, precision and recall are inversely related. As a search filter expands to collect more data, the proportion of relevant data decreases. Publications can present a precision-recall curve to show the extent of this trade-off for their search filter. A good search filter will maintain a relatively high level of precision as recall increases. A total of 15 articles reported precision, which ranged from 49.1% to 100% (Table 2). A total of 4 articles reported recall, which ranged from 86.6% to 99% (Table 2).

### Coding Methods

Coding methods for the studies included hand-coding, machine learning, or a combination of the two. Hand-coding involves one or more human coders categorizing data. When 2 or more coders independently code data, a coding agreement score (eg, kappa) between the coders can be calculated. In contrast, machine learning uses an algorithm for a computer to learn how to code data. However, human-coding is used for an initial subset of data to help refine the algorithm to improve its accuracy. Coding categories may be determined a priori based on prior research or they may be developed inductively through the process of coding. Studies used hand-coding only (n=17), machine learning + hand-coding (n=8), and machine learning only (n=2; Table 3). Of the studies using hand-coding, data were coded by the reviewed studies’ researchers in 16 studies, while 1 study used crowdsourcing (ie, many Web workers) [30]. All the studies that used machine learning also used initial hand-coding for a subset of the data, except for 2 studies that used topic modeling [31,32]. Topic modeling produces thematically related word clusters from the text [31].

The data collected in the hand-coded studies ranged from a collection period of 1 day to 1 year, while the machine learning studies ranged from 1 month to 5 years of Twitter data. Across the articles that used hand-coding, the number of coders per tweet ranged from 1 to 6. Coding agreement was reported by 20 articles: coding agreement percentage (n=5; 72% to 95.7%), kappa scores (n=13; kappa=.64 to 1.00), Cronbach alpha (n=1; alpha=.61 to 1.00), and both coding agreement percentage and kappa scores (n=1). Kappa values can be interpreted as poor (<.20), fair (.21-.40), moderate (.41-.60), good (.61-.80), and very good (.81-1.00) [51]. Cronbach alpha can be interpreted as unacceptable (<.50), poor (.50-.59), questionable (.60-.69), acceptable (.70-.79), good, (.80-.89), and excellent (>.90) [52]. However, it is important to note that these thresholds are not derived statistically but instead rely on intuitive judgments. The number of tweets coded per study ranged from 171 to 17,098 for hand-coding and from 7362 to 1,669,123 for machine learning. A total of 14 articles included retweets in their total number of tweets. A total of 15 articles reported the number of unique Twitter accounts, which ranged from 2 to 3804 for hand-coding studies and from 23,700 to 166,857 for machine learning studies (see Table 3).

URLs in tweets can provide information that changes the context or meaning of a tweet. Following URLs to their respective webpages can be time-consuming, but it can increase coding accuracy. Machine learning algorithms can analyze the text within URLs but may require human coders to follow them to their respective webpages. A total of 15 articles reported whether they followed URLs (followed: n=10, did not follow: n=5; Table 3). One article provided the most common URLs [26]. The studies tended to show that advertising or commercial tweets were significantly more likely to contain URLs than other types of tweets [25,33,34].

**Table 3:** Coding methods.

Article	Coding method	No. of coders	No. of tweets coded	Coded retweets	No. of Twitter accounts	Followed URLs	Coding agreement
[8]	Hand-coded by researchers	1: all tweets; 2: subsample 300 tweets	2248: relevance; 2192: content	Yes	NR^a^	No	91%: sentiment; 72%: theme
[25]	Hand-coded by researchers	6: for a subset of 250 tweets; NR for total	17,098: relevance; 10,128: content	Yes, if additional context	NR	Yes	κ=.64 to .70
[26]	Machine learning with initial hand-coding; Python Scikit-Learn	NR	1,669,123	Yes	NR	Yes	NR
[27]	Machine learning and hand-coding; naïve Bayes, k-nearest neighbors, and support vector machines	2: pilot of 1000; 2: random subset of 150; 2: all 7362	7362: relevance; 4215: content	Retweeted posts were only included once	NR	NR	κ>.70 for the random subset of 150
[28]	Hand-coded by researchers	1: all tweets; 2: for 10% subsample	300: complete sample; 300: industry-free sample; 481 of 600: content (duplicates between samples removed)	Yes	148: complete sample; 215: industry-free sample	Yes	κ=.74
[29]	Hand-coded by researchers	2	NR	Yes	Approximately 3400	NR	NR
[30]	Crowdsourcing with initial hand-coding	3	5000: relevance; 4978: content	NR	3804	NR	κ=.66 to .85 among a subset coded by researchers
[31]	Topic modeling with machine learning; MALLET, a command-line implementation of latent Dirichlet allocation (LDA)	NR	319,315: total; 95,738: hookah; 22,513: cigar; 201,064: cigarette	NR	NR	NR	NR
[32]	Topic modeling (LDA) with machine learning	NR	4962	NR	NR	NR	NR
[33]	Machine learning and hand-coding; DiscoverText	2: for a subset of 500 for relevance, 4500 for commercial versus organic, 7500 for cessation	73,672	Yes	23,700	Yes, hand-coded tweets with URLs	κ=.87 to .93
[34]	Hand-coded by researchers	1: all; 2: for subsets of 100 tweets	5000: relevance; 2847: content	NR	NR	Yes	κ=.64 to 1.00
[35]	Hand-coded by researchers	1: all tweets; 3: subsample	133	No	NR	NR	alpha = .61 to 1.00
[36]	Hand-coded by researchers	3	3935: relevance, foreign language, retweets; 2656 sampled for 288 original tweets for coding	No	346	Yes	κ=.64 to .91
[37]	Hand-coded by researchers; wordcloud R package	NR	171: relevance; 84: content	NR	84	NR	NR
[38]	Hand-coded by researchers	1: all tweets; 2: for 20% of tweets	143,287: identified; 4753: coded for clinical practice guidelines for treating tobacco dependence	NR	153	Yes	>90%
[39]	Hand-coded by researchers	2	684	Yes	306	Yes	NR
[40]	Machine learning and hand-coding; naïve Bayes, LIBLINEAR, Bayesian logistic regression, random forests; keyword comparisons	1: all tweets; 2: subsample of 2000	13,146	NR	2147	No, removed URLs	κ=.87 for subsample
[41]	Machine learning and hand-coding; human detection algorithm; Hedonometrics; key phrasal pattern matching	2: for all tweets from 500 automated accounts and 500 organic accounts as classified by the algorithm; 2: for 4 groups of 500 randomly sampled tweets to gauge accuracy of subcategorical tweet topics	850,000	Yes	131,622: automated accounts; 134717: organic accounts: 188,182: not classified accounts (ie, accounts with <25 tweets)	No, but the algorithm used the count of URLs to distinguish automated accounts from organic accounts; also used keywords in the URLs for the algorithm to determine subcategories of automated accounts	94.6% true- positive rate, 12.9% false- positive rate for the machines on the tweets from the 1000 accounts also coded with human-coding
[42]	Machine learning with initial hand-coding; Python Scikit-Learn; topic modeling with MALLET	2: for a subset of 1000 profiles	224,000 in 2013 sample; 349,401 in 2015 sample	Yes	34,000 in 2013 sample; 100,000 in 2015 sample	No; metadata on the presence of URL links	κ=.88
[43]	Hand-coded by researchers and MySQL pattern matcher	NR	1180	Yes	2: Blu and V2; 537: users retweeting Blu and V2	NR	NR
[44]	Hand-coded by researchers	1: all tweets; 2: for 20% of tweets (n=358)	2191: relevance; 1790: content	Yes	NR (>21)	NR	κ=.95 for 20% subsample
[45]	Machine learning with initial hand-coding; naïve Bayes classifier, k-nearest neighbors, support vector machines	6: for a subset of 250 tweets; NR for total	17,098: relevance; 10,128: content	Yes, if additional context	NR	NR	κ=.64 to .70
[46]	Hand-coded by researchers	3	1776	No	16	Yes	For 5% of data, 95.7%; κ=.72
[47]	Machine learning with initial hand-coding; naïve Bayes classifier	2: subset of 450 tweets for relevance; 2: subset of 350 tweets for content	245,319: relevance; 193,491: content	NR	166,857	NR; metadata on the presence of URL links	κ=.93
[48]	Hand-coded by researchers	1: all tweets; 2: for 1% of tweets	8645: relevance; 6257: content	Yes	NR	Yes	90% for a 1% sample of tweets
[49]	Hand-coded by researchers	2	900, with 50 tweets per account	Yes	18	NR	84%
[50]	Hand-coded by researchers	2	1519	No	1321	Yes	κ=.84

^a^NR: not reported.

### Coded Categories

All the studies developed categories for content. These content areas included one or more of the following: sentiment, theme, location of use, user description, profile photo, or location of user (Table 4).

#### Sentiment

A total of 9 articles coded for sentiment (Table 4). One article made a distinction between coding for sentiment (ie, emotional tone or affective content: positive, negative, or neutral) and message attitude (ie, pro, con, neutral or do not know) [28]. Two articles coded for sentiment in terms of emotional tone. In 6 articles, sentiment was described in terms of being supportive or against tobacco, tobacco users, or decisions regarding tobacco, which suggests an assessment of message attitude. Furthermore, 1 article assessed valence, but it was not clear whether positive or negative valence suggested an attitude or emotional tone [35].

#### Topic or Theme

A total of 21 studies coded for topic or theme (Table 4). The most common themes included the following: advertisement, marketing, industry or commercial (n=12 articles); health, safety, harms (n=9); use (n=8); policy, government, regulation, activism (n=7); e-cigarettes for smoking cessation (n=7); flavors (n=7); personal opinion or communication (n=6); risky behaviors or other substances (n=6); cessation (n=5); information (n=5), and craving or need (n=5). One study coded for location of use with 20 categories (eg, school, work) [26]. Finally, 2 studies used topic modeling to explore tobacco content [31,32].

#### User or Account

A total of 10 studies coded for user description from data found in the user profile, including type of account, age, location, and other characteristics (Table 4). The most common types of user categories coded were personal accounts (n=7 articles), industry accounts (n=5), news (n=5), unclassified (n=5), and bots, automatic, or fake (n=4; ie, automated computer program). None of the articles reported the percentage of tweets that had accounts without user profile information. However, across the articles with a category for tweets with an unclassifiable user description, 0.2%-38% of tweets were unclassified. Across the studies that coded for bots, the percentages of tweets varied drastically from 6.9% to 80.7%.

One study coded Twitter profile photos with 4 categories: gender, age, race, and single person versus multiple people [36]. Visual cues (eg, skin color, background themes, facial features) in the profile pictures were used for coding. Coding for age based on available visual cues in the profile photos tended to be difficult, so coding for age was simplified to 3 broad groups: young, middle age, and older adult. There were few middle-aged and older adults represented in the sample, so a dichotomous variable of young or not young was created [36].

A total of 4 studies coded for the location of the user with one each coding for state (California vs other) [8], country [37], continent [38], and city, state, and country (United States vs other) [39]. Location was identified for 51% to 63% of Twitter profiles. Most accounts that listed a location were from the United States and North America.

**Table 4:** Coded categories.

Category type	Category		Number of articles and percent of total^a^ n (%)	Articles
Relevance	Relevant versus nonrelevant		16 (59)	[8,25,27,30,33,34,36,37,41-45,47-49]
Sentiment			9 (33)	[8,25,27,28,35,41,45,48,50]
	Positive or negative (ie, supportive or against)		6 (22)	[8,25,27,45,48,50]
	Positive or negative (ie, emotional tone)		2 (7)	[28,41]
	Positive or negative valence		1 (4)	[35]
	Neutral or unknown		6 (22)	[8,27,28,45,48,50]
Message attitude	Pro or con		1 (4)	[28]
Type of utterance	Comparison versus attribution versus metonymy		1 (4)	[35]
Topics, themes, or genres			21 (78)	[8,25-28,30,33,34,36-43,45-47,49,50]
	Joke or humorous		3 (11)	[27,28,36]
	Song or music		2 (7)	[30,36]
	Profanity		1 (4)	[36]
	Social relationships		2 (7)	[27,50]
	Sex or romance		1 (4)	[30]
	Image or stereotype		1 (4)	[27]
	Risky behaviors or other substances		6 (22)	[25,27,28,30,36,45]
	Illicit substance use in e-cigarettes		2 (7)	[25,45]
	Preference for another substance		1 (4)	[30]
	Affiliation and preference		1 (4)	[36]
	Flavors		7 (26)	[25,36,39,41-43,45]
	Pleasure		1 (4)	[27]
	Tastes good		1 (4)	[28]
	Craving, desire, and need		5 (19)	[25,27,28,36,45]
	Addiction		1 (4)	[37]
	Type of tobacco product		4 (15)	[27,30,34,38]
	Type of tobacco product brand		1 (4)	[26]
	E-cigarettes’ smoke-free aspect		1 (4)	[42]
	Health, safety, harms		9 (33)	[8,25,27,30,33,37,39,45,50]
	Downplayed or refuted harms, harm reduction		2 (7)	[27,42]
	E-cigarettes for smoking cessation		7 (26)	[25,28,33,40-42,45]
	Cessation		5 (19)	[27,30,37,41,46]
	Cessation product		2 (7)	[34,38]
	Socioemotional support tweets regarding quitting smoking		1 (4)	[49]
	Encouraging or engaging tweets regarding quitting smoking		1 (4)	[49]
	Clinical practice guidelines for treating tobacco dependence		1 (4)	[38]
	Demonstration		1 (4)	[36]
	**Use**		8 (30)	[25,27,28,30,36,37,40,45]
		Use: general	2 (7)	[36,40]
		First-person use or intent	5 (19)	[25,27,28,30,45]
		Second- or third-person experience	4 (15)	[25,27,28,45]
		Starting use or smoking initiation	3 (11)	[27,28,37]
		Recent use	1 (4)	[30]
		Underage use	3 (11)	[25,27,45]
		Parental use	2 (7)	[25,45]
		Does not use or does not want to use	1 (4)	[30]
	Secondhand smoke		1 (4)	[46]
	Rejection and prevention		1 (4)	[36]
	Disgust, unattractive, or uncool		2 (7)	[27,30]
	Policy, government, regulation, activism, politics		7 (26)	[8,25,27,28,39,45,46]
	Normalization versus discouragement		1 (4)	[30]
	Getting others started or advocating use		1 (4)	[28]
	Attempt to engage other Twitter users		1 (4)	[28]
	Fear appeals		1 (4)	[47]
	Lies or propaganda		2 (7)	[8,39]
	Advertisement, promotion, marketing, industry, commercial		12 (44)	[8,25-28,30,33,36,38,41,45,50]
	Offering advice		1 (4)	[28]
	Personal opinion or communication		6 (22)	[25,27,28,33,38,45]
	News or update		4 (15)	[25,27,28,45]
	Information		5 (19)	[25,27,28,45,49]
	Science or scientific publication		2 (7)	[37,39]
	Cultural reference		1 (4)	[27]
	Issue salience		1 (4)	[39]
	Commodity		1 (4)	[27]
	Connoisseurship		1 (4)	[27]
	Cheaper than smoking		1 (4)	[28]
	Money		1 (4)	[8]
	Price promotion, discount, coupon		4 (15)	[26,33,34,41]
	Backgrounded		1 (4)	[27]
	Other or undetermined		2 (7)	[36,50]
Domains smoking was compared with for campaign slogans	Personal features; hobby or hype; person or group; social norm; big event; technology and innovation; sex or relation; eating, drinking, and stimulants; school; transport; and campaign		1 (4)	[35]
Links (URLs)	Most common links		1 (4)	[26]
Location of use			1 (4)	[26]
	Class		1 (4)	[26]
	House, room, bed		1 (4)	[26]
	School		1 (4)	[26]
	Public		1 (4)	[26]
	Bathroom		1 (4)	[26]
	Work		1 (4)	[26]
	In front of someone		1 (4)	[26]
	Car		1 (4)	[26]
	Restaurant		1 (4)	[26]
	Movie theater		1 (4)	[26]
	Airplanes or airport		1 (4)	[26]
	Store		1 (4)	[26]
	Bars or clubs		1 (4)	[26]
	Dormitory		1 (4)	[26]
	Library		1 (4)	[26]
	Mall		1 (4)	[26]
	Bowling alley		1 (4)	[26]
	Café or coffee shop		1 (4)	[26]
	Hospital		1 (4)	[26]
	Locker room		1 (4)	[26]
Topic modeling			2 (7)	[31,32]
	Hookah topic 1: social locations, leisure time, and positive affect		1 (4)	[31]
	Hookah topic 2: fun, leisure time, and sociability		1 (4)	[31]
	Cigarette topic 1: death and unpleasant smell		1 (4)	[31]
	Cigar topic 1: positive affect and enjoyment		1 (4)	[31]
	Cigar topic 2: luxury alcohol products		1 (4)	[31]
	Tobacco topic 1: tobacco use and substance use		1 (4)	[32]
	Tobacco topic 2: addiction recovery		1 (4)	[32]
	Tobacco topic 3: addiction recovery and tobacco promotion by clubs or bars		1 (4)	[32]
	Tobacco topic 4: tobacco promotion by bars or clubs and marijuana use		1 (4)	[32]
	Tobacco topic 5: antismoking and addiction recovery		1 (4)	[32]
User or account			10 (37)	[8,25,28,29,34,37,41,44,45,49]
	Government		3 (11)	[25,44,45]
	Foundations or nonprofit organizations		4 (15)	[25,44,45,49]
	Public health and health care		1 (4)	[28]
	Researcher or research center		2 (7)	[29,37]
	**News**		5 (19)	[25,28,37,44,45]
		Reputable news source	2 (7)	[25,45]
		Press, media, or news	3 (11)	[28,37,44]
		Medical news source	1 (4)	[37]
	**Personal accounts**		7 (26)	[8,25,28,29,37,44,45]
		Personal accounts, everyday people, individuals	6 (22)	[8,25,28,37,44,45]
		Personal accounts with industry ties	1 (4)	[28]
		Person: supporter	1 (4)	[29]
		Person: basic profile (no mention of e-cigarettes)	1 (4)	[29]
	Celebrity, public figures		3 (11)	[25,28,45]
	Organic (human)		1 (4)	[41]
	E-cigarette community movement		2 (7)	[25,45]
	**Industry**		5 (19)	[25,28,29,34,45]
		Industry: retailer or manufacturer	2 (7)	[28,29]
		Retailer or vendor	3 (11)	[25,34,45]
		Tobacco company	2 (7)	[25,45]
		Industry: other (eg, vaping magazine, Web marketer)	1 (4)	[29]
	For-profit organization		1 (4)	[44]
	Entity: general (eg, company, store, advocacy group)		1 (4)	[8]
	Nonperson (eg, musical band)		1 (4)	[29]
	Bots, automatic, fake		4 (15)	[25,28,38,41,45]
	Unclassified or other		5 (19)	[8,29,37,44,49]
Profile photo			1 (4)	[36]
	Single person versus multiple people		1 (4)	[36]
	Gender (male, female, mixed group)		1 (4)	[36]
	Age (babies or children, high school or college, adult)		1 (4)	[36]
	Race (African American, white, Hispanic, Asian, undetermined)		1 (4)	[36]
Location of user			4 (15)	[8,37-39]
	City, state, and country		1 (4)	[39]
	State		1 (4)	[8]
	Country		1 (4)	[37]
	Continent		1 (4)	[38]

^a^Percentages are rounded to the nearest whole percent.

## Discussion

### Overview

Studies analyzing tobacco-related Twitter data have grown in number in recent years. Although we searched for articles published from 2006 to 2016, articles meeting inclusion criteria for this review were published from 2011 to 2016, with 85% (23/27) of the publications occurring in 2014-2016. Widely used among adolescents and black and Hispanic individuals, Twitter could provide a rich source of tobacco-related data among these groups. One of the benefits of Twitter research is the ability to focus on emerging issues and products that are not yet addressed in surveillance or epidemiological research. Collecting tobacco data in real time via Twitter could be a useful tool for tobacco surveillance, which could help inform tobacco control policies and social media campaigns.

### Data Collection Methods

Twitter data can change rapidly as they are being posted daily [37]. If a study only collects data from one point in time, it may not be reflective of data at any other point in time. Twitter studies should consider collecting data at multiple time points or over longer periods of time to decrease the likelihood that results are idiosyncratic to that point in time. However, there may be some research questions that only require sampling one time or sampling directly before and after an event to gauge short-term responses.

The primary sources of data were Gnip and Twitter’s Streaming API, which offer different strengths and weaknesses. If a study wants access to all tobacco-related tweets, then Gnip may be more effective. However, if a study is interested in tweets about a specific tobacco campaign or has a constrained budget, then Twitter’s Streaming API may be a better data source. Alternatively, if a study is focusing on multiple social media sites (eg, Twitter, Facebook), then using Radian6, Simply Measured, or Sysomos Heartbeat may be appealing because of their cross-platform analysis.

The results of this review suggest that there are some gaps in the types of tobacco products studied by Twitter analyses. E-cigarettes were the product that was addressed by most studies, while none of the studies focused on smokeless tobacco, snus, bidis, or kreteks. The focus on e-cigarettes over other products could be due in part to their recent rise in popularity and recent debates about policies. From 2011 to 2014, e-cigarette use among high school students in the United States significantly increased from 1.5% to 13.4% [53]. Similarly, there was a significant increase in hookah use from 4.1% to 9.4%. However, there were significant decreases for cigarette (15.8% to 9.2%), snus (2.9% to 1.9%), cigar (11.6% to 8.2%), pipe (4.0% to 1.5%), and bidi (2.0% to 0.9%) use [53]. It could be useful to conduct studies comparing tweets about cigarettes, e-cigarettes, and hookah among adolescents to help understand the changing rates of use and Twitter postings that discuss issues related to use. The rates of e-cigarette, hookah, and cigar use among high school students may be affected by the FDA regulations of these products that went into effect on August 8, 2016. One of the provisions of these regulations is that it will be illegal to sell e-cigarettes, cigars, and hookah tobacco to persons younger than 18 years [54]. Researchers may want to analyze e-cigarette, cigar, and hookah tweets before and after August 8, 2016, to gain real-time insight into adolescents’ reactions to the new regulations.

Future Twitter studies could benefit from a standard of reporting data collection methods. Only 4 of the articles in this review reported such rigorous methods of selecting their search keywords as suggested by Kim and colleagues’ [11] search filter framework, while 15 articles reported retrieval precision and 4 reported retrieval recall. The terminology around newer products such as e-cigarettes is growing, so it may be difficult to capture all relevant Twitter conversations with one’s keywords [25]. Future studies will need to continue to refine and expand search keywords.

### Data Coding

The methods of coding were hand-coding, machine learning, or a combination of the two. Machine learning can code larger quantities of data at a quicker rate than hand-coding, but human coders may have greater discretion at coding for the complexities and subtlety of language such as humor, irony, or sarcasm. For example, algorithms developed to detect irony only retrieved 54%-57% of tweets coded as irony by multiple independent human coders [55]. Hand-coding can be subject to bias, but creating coding schemes based on prior literature and working to achieve acceptable levels of interrater reliability can help attenuate individual bias. Hand-coding allows researchers to follow URLs, which can change the meaning of the tweet. Viewing the webpage may provide additional information that may not be discernable from the URL. Studies that require determining subtle differences in context may be better suited to hand-coding a small sample of Twitter data, while studies that rely less on context could code large samples with machine learning.

### Coded Categories

The sentiment of tweets could help evaluate whether the responses to pro- and antitobacco efforts are positive or negative as a way of understanding social norms about these products. Clarity and comparability across studies could be improved if a distinction is made between attitude and emotion when coding for sentiment. For example, a tweet could be pro-vaping but have a negative emotional tone or it could be anti-vaping but have a positive tone. Only 1 article clarified the meaning by making an explicit distinction between coding for emotional sentiment and message attitude [28]. This is reflective of coding for stance (in favor of, against, or neutral) versus sentiment (emotional tone) [56].

The three most common themes used for coding were advertisements or marketing; health, safety, harms; and use. Surveillance of these themes could be beneficial to understand whether tobacco advertisements are being circulated on Twitter with the potential of reaching underage individuals, whether the content is making unproven claims about the health and safety of their products, or promoting the use of their products to vulnerable populations such as youth or ethnic minorities.

The most common user account descriptions that the studies coded for were personal accounts, industry, news, unclassified, and bots. Determining the user description and demographic information for the accounts that tweet about tobacco could help determine whether tobacco companies, pro-vaping advocacy groups, or antitobacco efforts are circulating with a potential to reach certain groups (eg, adolescents). Researchers could also monitor how news organizations are presenting tobacco-related information to the public.

It may be difficult to determine the demographics of the person tweeting or of the audience exposed to the tweets, which could be especially problematic when studies want to focus on vulnerable populations (eg, adolescents). A Twitter account could be run by an individual, multiple people (eg, vape shop employees), or bots. To reduce bias, bots should be identified and the tweets from these accounts removed from analysis or identified as tweets originating from automated accounts [57]. Only 4 studies coded for bots with a range from 6.9% to 80.7% of tweets classified as bots. Even if an account is run by an individual, a Twitter profile provides little information. It may or may not include a photograph, profile description, location, website, and birthday. It does not include gender or ethnicity and race, so this information needs to be estimated. Although none of the studies reported the percentage of tweets with accounts missing profile information, 5 studies coded for an unclassified category with 0.2%-38% of tweets being unable to be classified based on the account profile. If profile information is included, it could be used in combination with natural language processing to infer information about the individual from his or her tweets [29]. For example, algorithms have estimated gender with a 75.5% accuracy based on tweets and a 92.0% accuracy based on tweets, screen name, full name, and profile description [58]. A study that combined analysis of text and image processing predicted gender with an accuracy of 85.1% [59]. Additionally, algorithms based on tweets were capable of predicting the exact age of the user within a margin of 4 years, while accuracy for age categories were 93.0% for <20 years, 67.4% for 20-40 years, and 81.6 for >40 years [60].

### Recommendations

It is recommended that tobacco Twitter studies adopt methodological standards of reporting and data quality assessment. Important information to consider reporting include data sources (eg, Gnip, Twitter’s Streaming API), the date range of tweets collected, the number of tweets coded, whether retweets were coded, whether coders followed URLs, whether images were coded, the categories coded, the decision criteria for each category, the number of unique Twitter accounts, and the types of Twitter accounts. Studies that use hand-coding should also consider reporting the number of independent coders, the number of tweets coded across coders, and their coding agreement, while it is important for machine learning studies to detail the development and refinement of their algorithms. Providing this information is likely to increase comparability across studies and the ability to replicate results.

Depending on research goals, studies may want to sample Twitter’s databases multiple times, especially if they collect from Twitter’s Search API or Streaming API, which put limitations on the amount of data that can be collected per sample. Sampling at multiple times is also important for studies that have access to Twitter’s Firehose through Gnip, considering that tweets are in real time, which could lead to fluctuations based on real-world events (eg, the FDA’s new regulations for e-cigarettes). Following messages over time could also help establish trends in the content of posts. However, some studies may be interested in short-term reactions to an event, which requires different sampling methods than following trends. For example, if a study is interested in short-term reactions to the FDA’s new regulations for e-cigarettes, then a single sample before and after the implementation of the new regulations could be sufficient.

When coding for sentiment, researchers could improve clarity by making a distinction between whether they are coding for message attitude or for emotional tone [28]. A clear distinction between the two could improve comparability of sentiment ratings across studies.

If relevant to the research questions, Twitter studies may want to code images and URLs. This added step can be time-consuming, but doing so could change the context or meaning of a tweet. For example, an image could help determine if a tweet should be coded as humor or sarcasm, while following a URL could help determine if the tweet is an advertisement. Failing to code images and URLs could result in missing significant content that could affect coding accuracy and skew results.

Analyzing Twitter user profiles could provide context for tweets. The same pro–e-cigarette tweet could hold very different significance for a study’s results if it is from a vape shop versus an adolescent. However, the limitations of analyzing user profiles should be recognized and steps taken to improve accuracy. It should be noted that user profiles may be misleading (eg, a tobacco industry representative posing as an unaffiliated citizen) or profiles of bots. Bots should be identified and potentially removed to reduce bias and improve the quality of data [57]. We cannot assume that the analysis is describing individuals but must instead consider the poster to be an “account” rather than a person.

A limitation of Twitter data is that it does not provide much information on the effects of tweets on behavior. For example, tweeting about tobacco use does not necessarily mean that the person tweeting uses tobacco [40]. Additionally, an individual may tweet about a quit smoking campaign with positive sentiment, but that does not mean that the campaign has influenced his or her smoking behavior. Follow-up studies with the individuals tweeting about the campaign would need to be conducted. None of the studies in the review included follow-up survey studies with individuals from their Twitter sample. Although recruitment of individuals through Twitter may come with its own set of obstacles, this could be an avenue for future Twitter research. Following specific individuals over time could allow for the analysis of changes in message content.

### Review Limitations

There are several limitations of this systematic review. First, the results are limited to the databases and search keywords selected, which could have resulted in incomplete retrieval of identified research. Second, this review is limited to its inclusion criteria and the decision rules of the single, independent coder who selected the articles and extracted the data to be included in the review. Individual bias was limited by explicit inclusion, exclusion, and data extraction criteria. However, some studies that were included or excluded for this review may have varied given different inclusion criteria or a different coder. Finally, this review is limited to methodology of categorically coded tobacco Twitter data. Different methodological results and recommendations may have been made if the topic of Twitter data had been different (eg, marijuana) or if the review had focused on different outcomes (eg, popularity of tweets or diffusion of tweets).

### Conclusions

Categorically coded Twitter research can be used for certain insights that other survey research does not provide: emerging issues, popular content in real time, changes over time, how tobacco companies and pro-vaping advocacy groups use social media to increase message exposure in the population (eg, youth who might otherwise be protected from tobacco marketing), how tobacco control policies and campaigns can most effectively use social media, arguments by groups that may be incorporated into media message design, and quick reactions to antitobacco media campaigns and regulations. There are several approaches that researchers are taking to this end, each having its own set of strengths and weaknesses. Standards for data collection and coding should be developed to more easily compare and replicate tobacco-related Twitter results. Additional recommendations, dependent on one’s research goals, include the following: sample Twitter’s databases multiple times, make a distinction between message attitude and emotional tone for sentiment, code images and URLs, analyze user profiles, and identify and remove bots.

## Abbreviations

API: application programming interface
FDA: US Food and Drug Administration
